# Supplementary material for: Microbial Spectrum and Antibiotic Resistance in Patients Suffering from Penetrating Crohn’s Disease
Source: J Clin Med. 2022 Jul 26;11(15):4343. doi: 10.3390/jcm11154343 (PMC9330589; doi:10.3390/jcm11154343)

Supplemental Tables and Figures

Supplemental Table S1: Groups of pathogens.

**Viridans group streptococci**	*S. intermedius, S. anginosus, S. constellatus, S. pneumoniae, S. Savlivarius, S. mitis, S. parasanguinis, S. gordonii, Gemella spp., S. lagdunensis*
**Streptococcus groups A, B, C and G**	*S. pyogenes, S. agalactiae, S. suis, S. bovis, group F, S. alactolyticus, S. pseudoporcinus*
Staphylococcus spp.	*S. aureus*
**Anaerobic bacteria**	*Lactobacillus spp., Actinomyces Spp., Bifidobacterium spp., Peptostroptococcus spp., P. micra, Finegoldia magna, Clostridium spp., Bacterioides spp, B. fragilis, B. vulgatus, B. uniformis, B. caccae, B. ovatus, B. thetaiotaomicron, F. necrophorum, F. nucleatum, Parabacteroides ssp., Prevotella spp., O. splanchnicus, V. parvula*
Enterobacterales	*Enterobacter ssp., E. coli, E. fergusonii, Klebsiella ssp, K. pneumoniae, K. oxytoca, Salmonella ssp., Shigella spp., P. mirabilis, P. vulgaris, P penneri , Yersinia spp., Morganella morganii spp., Haemophilus parainfluenzae, coliforme germs, Citrobacter spp., R. planticola, Hafnia alvei*
Enterococcus spp.	*E. faecalis, E. faecium, E. hirae, E. avum*
**Non relevant**	*S. epidermidis, S. hominis spp., Corynebacterium spp., P. oryzihabitans, P. Aeruginosa, N. Gonorrhoeae*
**Fungi**	*C. albicans, C. glabrata, C. dubliniensis, S. cerevisiae*

Supplemental Figure S1: Local resistance profile for general surgery at the University Hospital of Wuerzburg (all materials; CIP (fluoroquinolone), ciprofloxacin; CTX, cefotaxime (third-generation cephalosporin)).

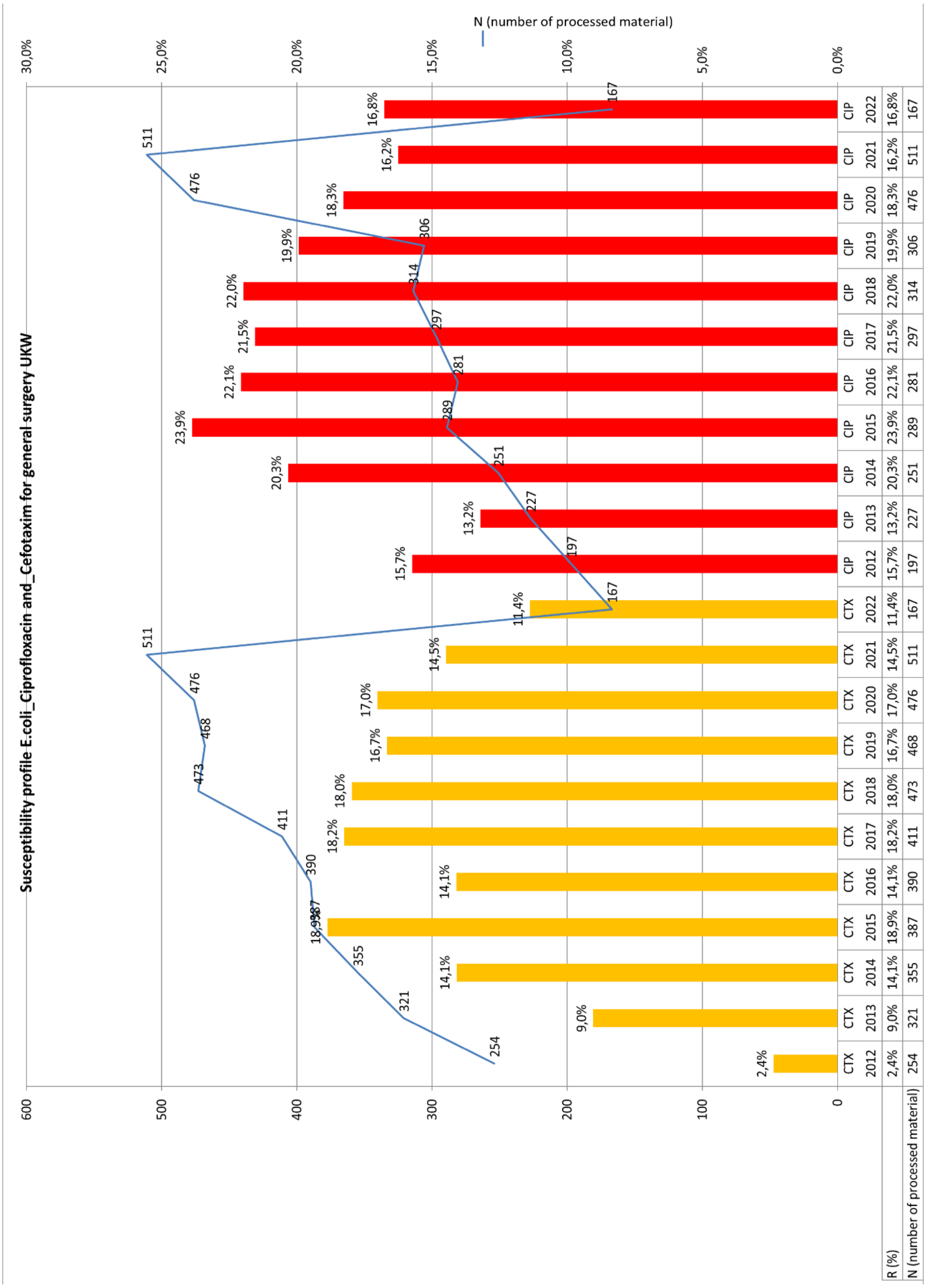

Supplemental Figure S2: Total antibiotic use in University Hospital of Wuerzburg, third-generation cephalosporins, 2016-2021 (Inf, infusion).

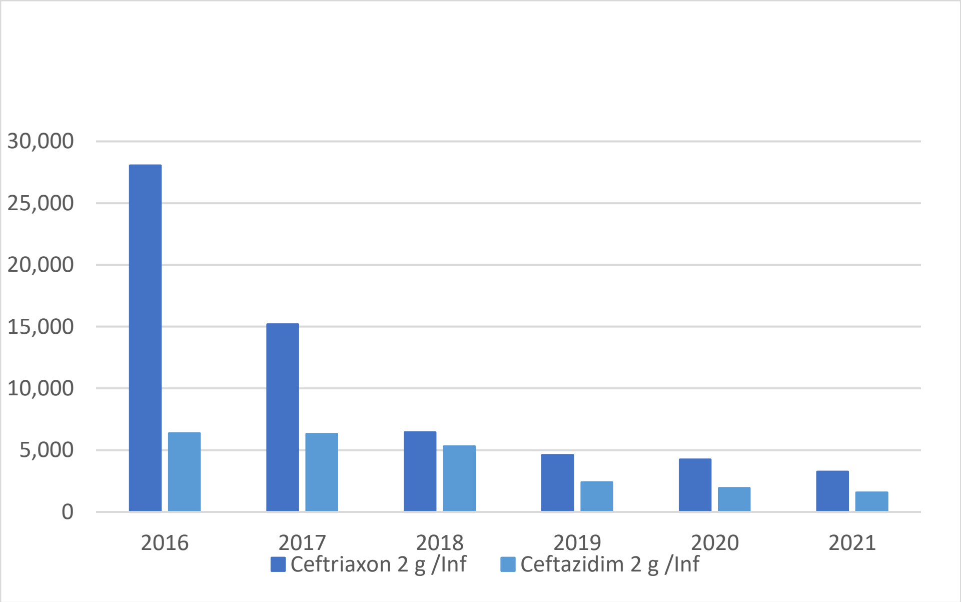

Supplemental Figure S3: Total antibiotic use in University Hospital of Wuerzburg, ciprofloxacine, 2016-2021 (Inf, infusion; Tbl, tablet).

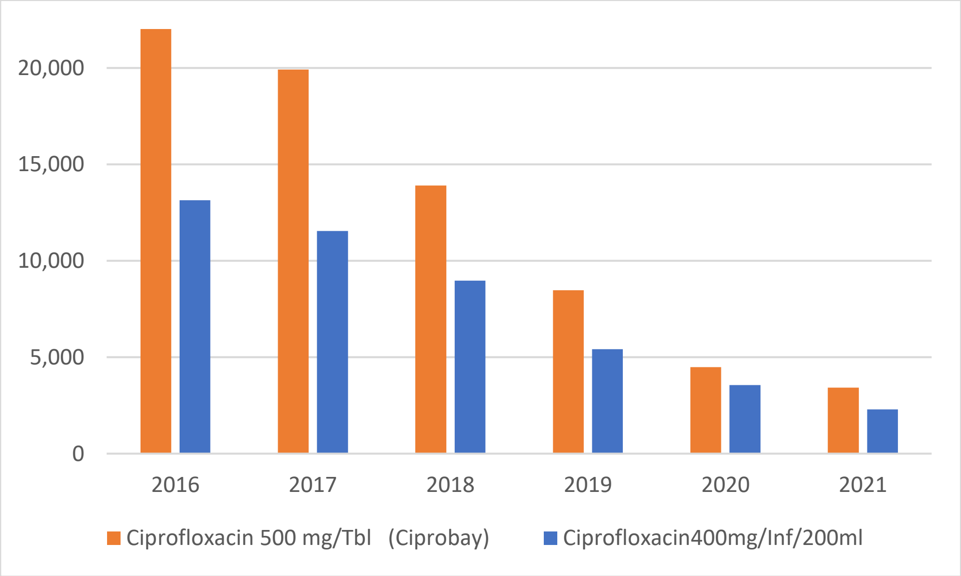

Supplement: Supplementary file 1 [file jcm-11-04343-s001.zip › jcm-1826909-supplementary.pdf]
